# Establishing a laboratory colony of the human flea, *Pulex irritans*: methods for collecting, rearing, and feeding

**DOI:** 10.1186/s13071-025-07001-9

**Published:** 2025-08-27

**Authors:** Adelaide Miarinjara, Annick Onimalala Raveloson, Mandimby Rajaonarimanana, Diego Ayala, Romain Girod, Thomas Robert Gillespie

**Affiliations:** 1 Departments of Environmental Sciences and Environmental Health, Emory University and Rollins School of Public Health, Atlanta, USA; 2 University of Antananarivo, Antananarivo, Madagascar; 3 Institut Pasteur de Madagascar, Antananarivo, Madagascar; 4 UMR MIVEGEC, University of Montpellier, CNRS, IRD, Montpellier, France; 5 Centre ValBio, Ranomafana, Fianarantsoa, Madagascar

**Keywords:** Laboratory rearing, Artificial feeding, Vector biology, Plague vector, Ectoparasite, Colony maintenance, Arthropod vectors, Entomology, Flea colony, Siphonaptera

## Abstract

**Background:**

Colonizing fleas under laboratory conditions is a crucial step to studying their biology, conducting bioassays, and evaluating their ability to transmit pathogens. Starting a colony implies collecting and maintaining wild-caught specimens to obtain the next generations. Here we describe methods to collect, safely transport, and maintain adult and immature stages, and for the first time, to produce viable next generations of *Pulex irritans*, the human flea in the insectary.

**Methods:**

Adult fleas were collected using human landing catches, while immature stages were obtained using the Berlese–Tullgren method. Blood feeding was performed using an artificial feeding device and the survival of adult fleas maintained on human or sheep blood was assessed.

**Results:**

More than 200 F0 adults survived and produced eggs for approximately 6 weeks, with an average lifespan of 6 days for males and 10 days for females. Pupation occurred around 10 days after arrival in the laboratory, yielding more than 900 cocoons within 8 weeks, with an emergence rate of approximately 80%. Challenges included high mortality among F1 adults, with both sexes surviving an average of 7 days. Although blood source assay was inconclusive, fleas were maintained on human blood. Factors that may have contributed to the low survival of F1 are discussed.

**Conclusions:**

This study provides a foundational framework for laboratory-based research on *P. irritans* and its role in vector-borne disease transmission. While further studies are needed to establish a sustainable laboratory colony, we demonstrate that a substantial F1 population can be obtained within 3 weeks of laboratory rearing, enabling experimental studies on this species.

**Graphical Abstract:**

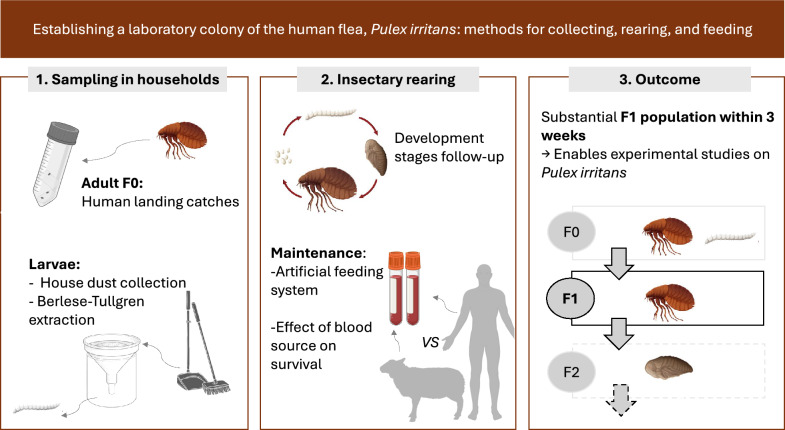

**Supplementary Information:**

The online version contains supplementary material available at 10.1186/s13071-025-07001-9.

## Background

Fleas are blood-feeding insects that can live on a large array of mammals and birds, however, each species usually have only a few potential hosts [1]. Among them, the rat flea *Xenopsylla cheopis* is well known as the primary vector of *Yersinia pestis*, the bacterium responsible for bubonic plague transmitted between rats and humans [2–4]. Other flea species are also closely associated with humans. In particular, the human flea, *Pulex irritans*, has a long history of association with humans and their immediate environment [5–7]. However, *P. irritans* is also known to be a parasite of wild animals such as foxes, badgers, and burrowing owls [7–13].

While often considered a minor vector of *Y. pestis*, *P. irritans* has been repeatedly detected during outbreaks, raising epidemiological concerns [14–17]. Experimental studies have shown that *P. irritans* is capable of transmitting *Y. pestis* to naïve hosts, though less efficiently than *X. cheopis* [18]. Challenges such as the reluctance of *P. irritans* to feed on laboratory rodents and high mortality during experiments complicate these studies, consequently limiting the observation of infection progression. [14, 15, 18–21].

Beyond plague disease, *P. irritans* remains an important ectoparasite of humans and livestock. Flea infestations can cause anemia, dermatitis, and allergic reactions in animals, particularly in young or vulnerable individuals. In sheep and goats, for instance, behavioral responses to *P. irritans* infestation, such as excessive grooming, may lead to skin damage, infections, and reduced productivity, ultimately resulting in economic losses [22–24]. In humans, similar discomfort and distress may drive excessive insecticide use, raising health and environmental concerns [25, 26].

These impacts have motivated experimental research into pest control [27–29] and vector competence [14, 18, 20, 21, 30]. However, such studies largely rely on wild-caught *P. irritans* (F0), which presents limitations such as unknown age and insecticide exposure history, variable physiological status, and inconsistent availability. These limitations may complicate the studies of *P. irritans* biology, vector competence assessment, or the monitoring of its susceptibility to insecticides [31]. A laboratory colony would help overcome these issues, providing standardized and healthy fleas for research. While methods for laboratory colonization of other flea species exist, notably *X. cheopis* [32–36], efforts to colonize *P. irritans* have not been sustained or replicated [30].

The aim of this study is to describe methods used to collect, transport, and maintain *P. irritans* from the field to obtain subsequent generations in the laboratory. Here we report and discuss the results of the methods we developed.

## Methods

### Collection technique

Fieldwork was conducted in August 2024, in the commune of Miantso, district of Ankazobe, situated approximately 90 km northwest of Antananarivo, the capital of Madagascar. The population is predominantly rural, with local communities relying on subsistence farming. Ankazobe district has been noted as a plague hotspot [37]. Both adult fleas and immature stages (eggs and larvae) were collected from households. All field-collected individuals, regardless of life stage, were classified as F0 generation. Fleas that emerged in the laboratory from these immature stages were considered F1.

Approximately 20 households were invited to participate, with those consenting instructed to collect adult fleas using one tube per night. The involvement of households was terminated if they collected less than four fleas during one capture session, otherwise, they continued the flea collection during three successive nights. After adult flea collection sessions, households with the highest number of adult fleas caught were selected for dirt collection containing the immature flea stages.

#### Adult fleas

**Fig. 1:**
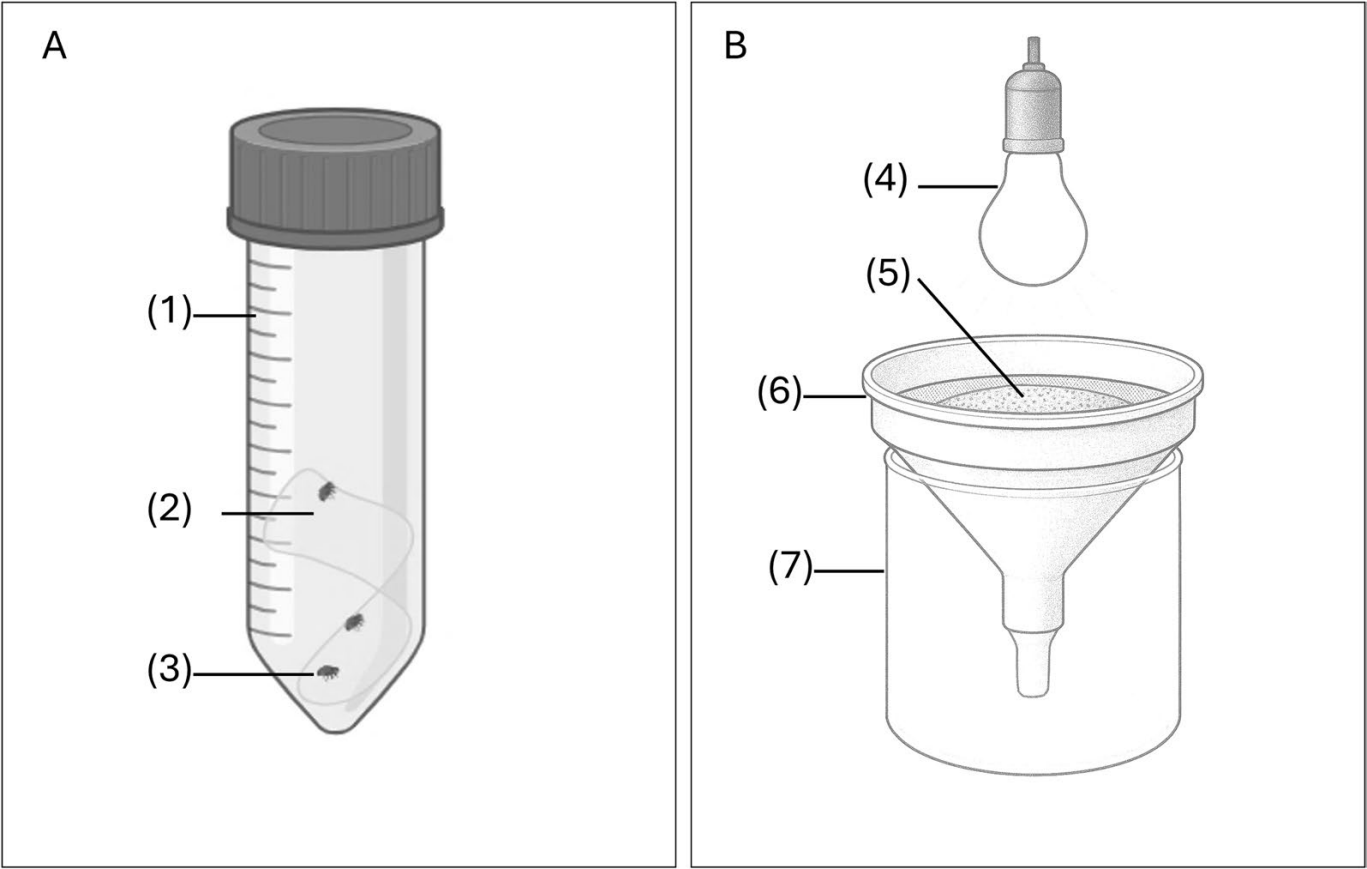
Diagram of the sampling tools. **A** Sampling tube: (1) 50 ml conical tube with a (2) strip of muslin cloth, which serves as foothold for (3) adult fleas. **B** Berlese–Tullgren device: (4) light bulb, (5) dirt, (6) strainer with sieve attached to a funnel, (7) plastic cup. The photo of the Berlese–Tullgren device is in Additional file 1: Fig. S1

Adult fleas were collected by homeowners primarily during the evening and the night. Some participants captured fleas when they felt them crawling on their bodies or biting them during their awake time. Some homeowners managed to catch fleas while being disturbed by flea bites during their sleep, while others reported collecting fleas from their bedsheets and clothing upon waking in the morning. None of them reported catching fleas on domestic animals. They used their index and thumb to catch the fleas and placed them into 50 ml conical centrifuge tubes that contained a strip of muslin at the bottom (Fig. 1A). The muslin strip helped retain the fleas and prevented them from jumping out immediately when the tube was opened again. In the morning, the tubes were collected, and households scheduled for the following night received new tubes. After collection, they were transferred from tubes into rearing containers and transported to the laboratory in a cooler. To ensure their survival until arrival at the insectary, collected adult fleas (F0) were fed daily using an artificial feeding system (described in adult feeding section).

#### Immature stages

To collect the immature stages in house dust, a car vacuum cleaner was used to collect dust on hard-to-reach surfaces (under heavy furniture) and a duster and dustpan were used to collect dust under mat at the corners and edge of the room. Dust samples were stored in resealable plastic pouches and transported to the field lab. To remove coarse debris, the dust was sifted using a #20 sieve (with 850 µm openings), with the larger particles discarded. Flea larvae present in the dust were then extracted using a Berlese–Tullgren (BT) funnel device [38], constructed from a large kitchen strainer connected to a funnel leading into a 500 ml white plastic cup (Fig. 1B, Additional file 1: Fig. S1). A 25-W lamp bulb was positioned about 12 cm above the inner surface of the strainer. Approximately 1 cm (layer thickness) of dust was placed in the strainer and exposed to the lamp for 4 h. The larvae, moving away from the light and heat of the lamp, were collected in the plastic cup below. After the exposure period, the flea larvae were transferred to a rearing medium in a plastic food container and transported into the insectary. Field-collected adults (F0) were maintained separately from immature stages collected with the BT device.

The larval rearing medium consists of an equal volume mixture of sand and sawdust and larval food [30, 39]. The sand and sawdust are sieved beforehand (sieve #20) and sterilized before use. Larval food is mixed with the rearing medium at a rate of 1 g per 50 g. Larval food is composed of 100 g sifted dog food powder (sieve #40, 425 µm openings), 15 g of sheep blood powder, 10 g of brewer’s yeast, and stored at 4 °C until use.

### Insectary rearing setup

#### Species identification

For morphological identification of species, alive adult fleas were immobilized using a combination of cold and carbon dioxide (CO₂) exposure. Fleas were released from rearing container into an 80 cm-deep tub and collected with a manual vacuum pump connected to a glass tube, which was then partially submerged in ice. Fleas inside were exposed to a low CO_2_ flow (BG-CO_2_ Generator, Biogents, USA) for approximately 5 min. For microscopic examination under a stereomicroscope, individuals were maintained in a Petri dish on a chill table kept below 0 °C. The species of field-collected adults (F0 generation) were morphologically identified upon arrival at the laboratory using a taxonomic key [40]. To prevent contamination with nontarget species from household dust, post-emergence identification of F1 adults was also performed.

#### Rearing unit design

**Fig. 2:**
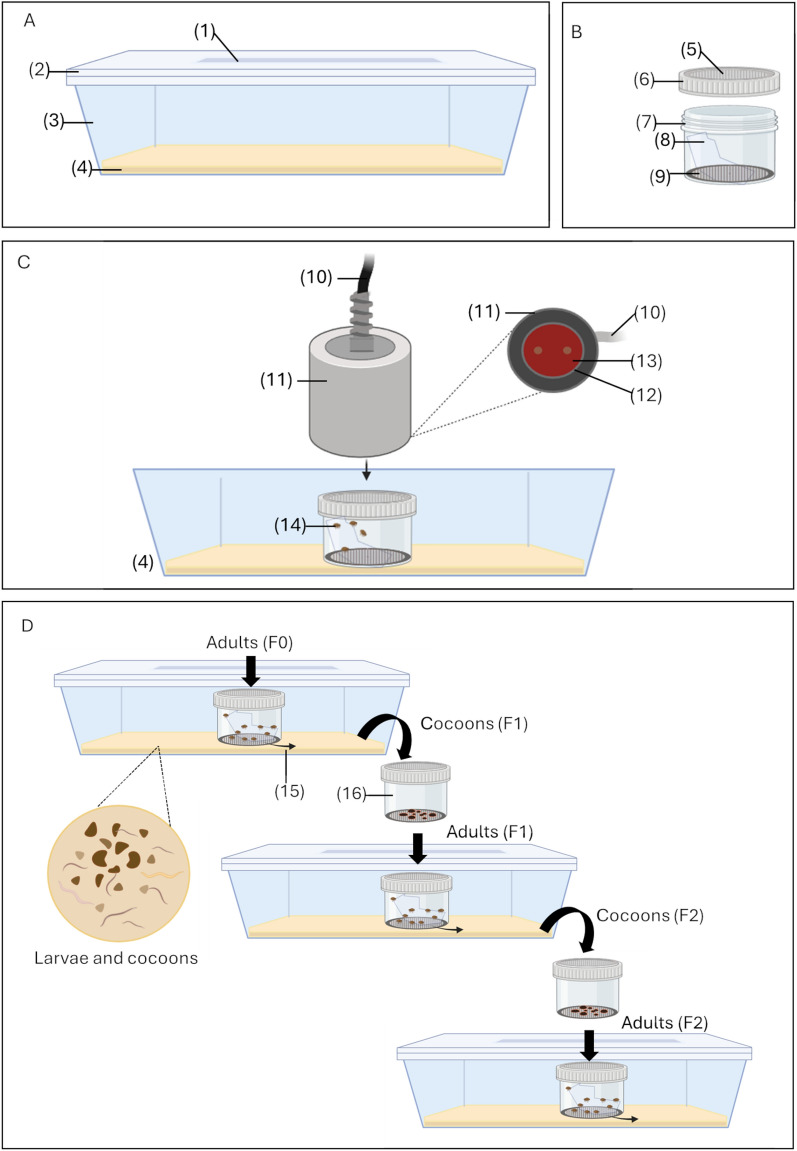
Diagram of the rearing and feeding containers. **A** Larvae container: (1) muslin glued to the carved lid, (2) lid, (3) plastic food tray, (4) larva rearing medium. **B** Adult container: (5) muslin glued to the carved cap, (6) cap, (7) plastic cup, (8) strip of muslin, (9) metal mesh. **C** Feeding setup: (10) power chord connected to Hemotek power unit, (11) feeder, (12) blood reservoir lined with stretched parafilm, (13) blood, (14) fleas. **D** Colony follow-up: (15) arrow indicates first instar larvae moving from adult container to larvae rearing medium, (16) pupae container. Photos of each element are included in the Additional file 1: Fig. S1

Adults and larvae were reared at the quarantine insectary at a temperature between 22 °C and 26 °C and a relative humidity between 60% and 85%. The rearing setup followed a two-part system inspired from Hudson & Prince pillbox in which the adult container is enclosed within the larval container [30]. The outer part, referred to as larval container (Fig. 2A), is made of translucent plastic food tray containing the larval rearing medium. To regulate moisture inside the rearing unit, a rectangular section was cut from the lid and replaced with a piece of muslin, secured by glue. The inner adult container (Fig. 2B) was a plastic cup with its bottom removed and replaced with a metal mesh disc, allowing for the hatched first instar larvae to enter the medium below, while keeping adults contained. A strip of muslin inside the adult container provided a foothold for the adult fleas. The lid of the cup was also modified with muslin to allow airflow and facilitate artificial feeding. All larvae in the two-part rearing system came from eggs laid under laboratory conditions and immature stages from BT device had their dedicated larval container.

#### Adult feeding

The adult fleas were fed with human blood from volunteers using an artificial feeder, Hemotek device (http://hemotek.co.uk/), where a parafilm-covered tank filled with blood was placed directly on top of the muslin-covered lid of the adult container (Fig. 2C). Blood was obtained weekly from volunteers and sheep using 5 ml venous blood collection tube coated with heparin and stored at 4 °C. Human blood was used exclusively for routine colony maintenance. For experimental purposes investigating the effect of different blood sources, sheep blood was also collected and used following the same protocol.

Adult fleas were fed daily for 5 h, either directly inside the rearing container or transferred in feeding container (Fig. 2C). Transferring the fleas to a new clean feeding container allowed for close observation of their feeding behavior. Well-fed *P. irritans* are known to excrete large amounts of undigested blood [32], which could be clearly seen as blotches of blood on the muslin or the inner surface of the container after feeding.

#### Colony follow-up

The first instar larvae that hatch from eggs laid in the adult container pass through the mesh into the larvae container, which contained approximately 0.5 cm thick of rearing medium (Fig. 2D). The number of dead adults were counted before the daily feeding. Once a week, the rearing medium was examined for larvae and pupae presence. Newly formed cocoons were moved to the pupae container (Fig. 2D) in which the adult will emerge. Once the adults emerged from the cocoons, they were moved to a new rearing container and a new cycle started. Species morphological identification can be repeated at this stage to avoid contamination with other flea species. While the cocoons are regularly removed from the larvae container, the remaining adults still lay eggs and cycles can occur inside a single rearing unit until the reproducing adult stock is depleted.

### Effect of blood source on survival

To assess the impact of blood source on adult survival and fitness, two groups were segregated from adults F1 and fed every day either with human (*n* = 44) or sheep blood (*n* = 32). The number of dead were counted daily, and survival was compared between the two groups. Since sheep blood is more easily accessible, we used F1 individuals that had never fed on human blood to gradually adapt the colony to feed exclusively on sheep blood.

### Statistical analysis

Statistical analysis was performed using R studio [41] with the survival and survminer packages. Kaplan–Meier curves were plotted to show the survival probability over time. Cox proportional hazards model was employed to evaluate survival differences between groups. The model estimates the hazard ratio (HR), which indicates the relative risk of death occurring in one group compared with another. Concordance index (C) indicates the model’s predictive accuracy.

## Results

### Adult flea collection yield

In total, 18 households agreed to participate in the study and a total of 299 adult fleas were obtained through three successive nights (Table 1). At least one flea was obtained from 10 households, which gives an average of 30 fleas per household.

**Table 1:** Number of F0

Household #	Night 1	Night 2	Night 3
1	5	0	–
2*	30	13	24
3	6	10	23
4	0	–	–
5	0	–	–
6	4	0	–
7	8	12	16
8	0	–	–
9*	10	7	18
10*	–	45	38
11	–	0	–
12	–	4	12
13	–	0	–
14	–	1	–
15	–	0	–
6	–	1	–
17	–	0	–
18	–	2	–
Other	–	–	6
Total	67	95	137

Adult fleas sampled per visited household, Miantso, Ankazobe district, Madagascar

^*^Households where dust was collected. “–” indicates that sampling was not performed that night

### Immature stage collection with Berlese–Tullgren device

**Fig. 3:**
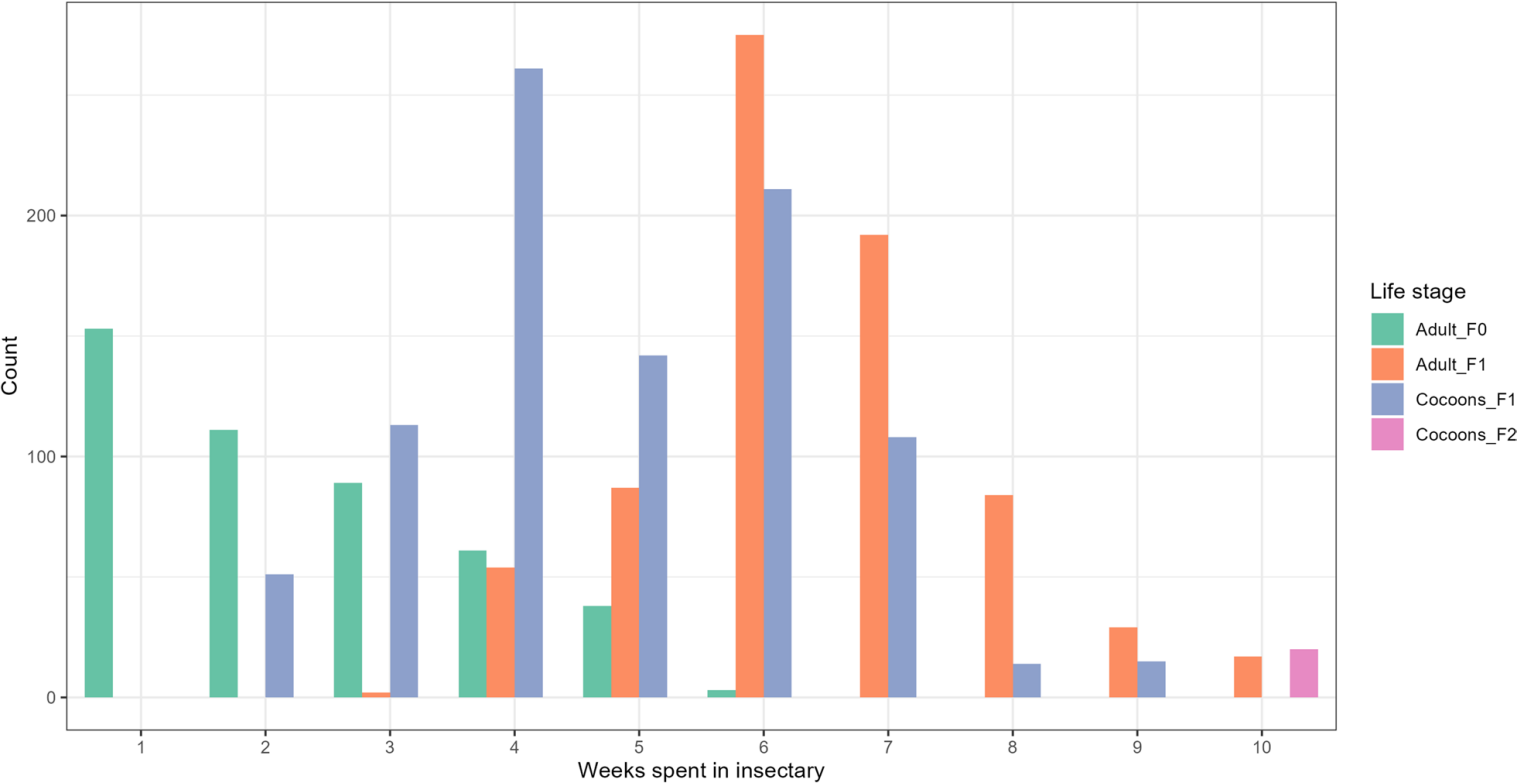
Weekly evolution of *P. irritans* cocoons (F1 and F2) and adults (F0 and F1) numbers following collection session. The number of cocoons is an estimate

Three households with the highest flea number agreed to dust collection in their homes. The first session of larvae extraction with the BT device was conducted in the field. About 1.5 kg of dust was processed and the extracted larvae (number not counted) were divided into four containers with rearing medium. The dust from which larvae was extracted was divided into rearing containers and brought into the laboratory for further extraction. During the first week in the insectary (week 1), the same dust was extracted again with the BT and yielded larvae of various stages that were reared in the insectary. After the last extraction, the dust was sterilized and discarded with laboratory waste. A total of 41 cocoons were obtained from these larvae containers after 2 weeks in the insectary (Fig. 3). During the third week, 28 additional cocoons were collected. By the end of the fourth week, a total of 75 cocoons were collected from the household dust using the Berlese–Tullgren device.

### Survival of adult F0

**Fig. 4:**
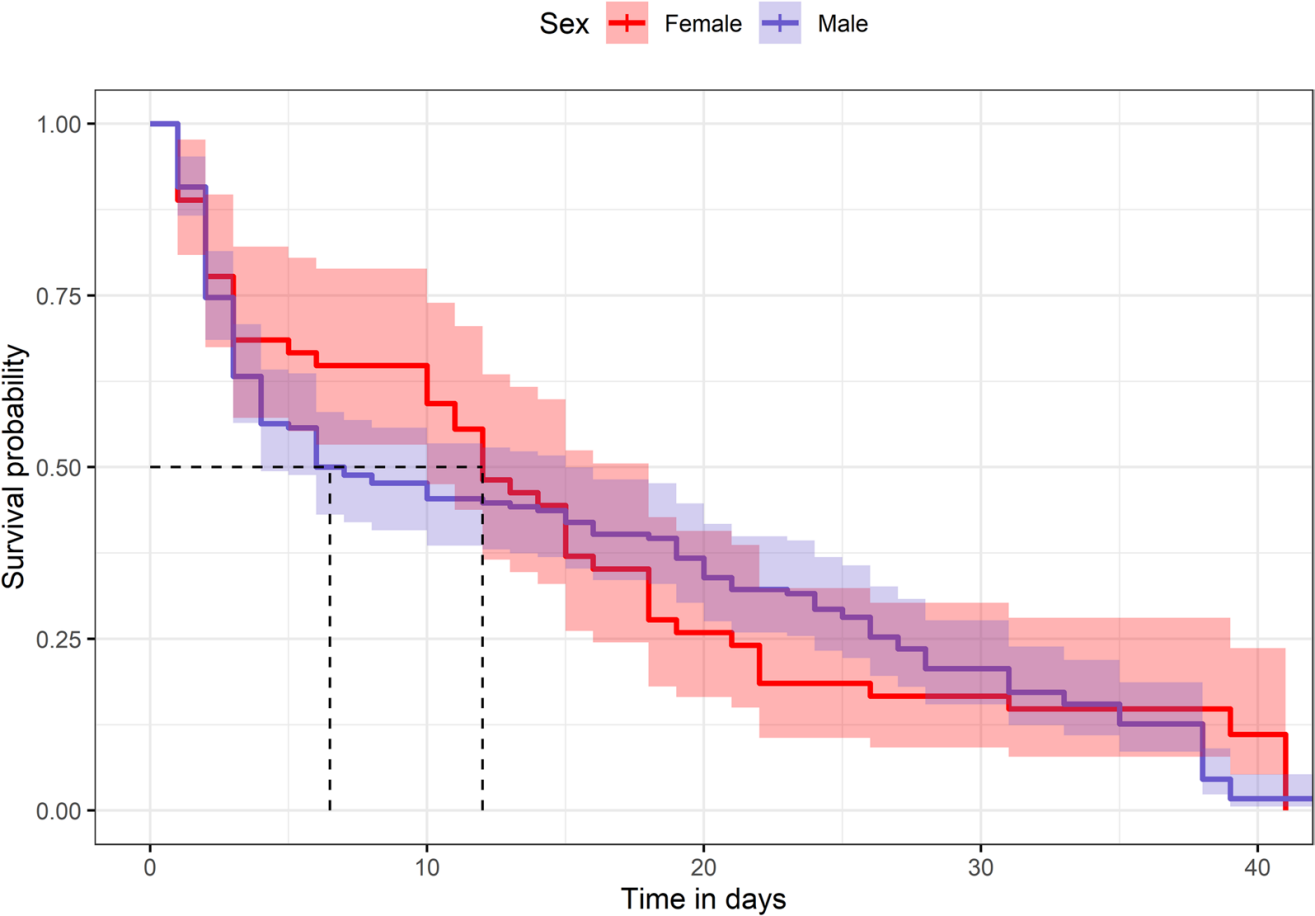
Survival curve of *P. irritans* males and females of the generation F0 in the insectary. The number of dead and alive *P. irritans* fleas for each group was merged. Dotted lines show the median survival for male and female. Time is in days spent in the insectary

Among the 299 fleas collected in the field, 235 (174 males and 61 females) were reared in insectary as F0 generation and divided into three containers (named group 1 to 3). The number of dead fleas per day ranged from 1 to 28 for males and 1 to 6 for females. Adults F0 survived about 6 weeks after arriving in the lab, with 50% of males dead within the first week, with a median survival of 6 days (95% CI 4–15 days), and females with a median survival of 10 days (95% CI 10–18 days). Although the female appears to survive longer according to median value, log-rank test considering the full breath of the survival curve (Fig. 4) showed that male and female F0 survival distributions are the same (*χ*_2_ = 0.2, df = 1, *P* = 0.6). The results from the Cox proportional hazards model indicate that sex did not have a statistically significant effect on survival. Specifically, HR for males is 1.097, suggesting that males have a slightly higher risk of death compared with females, but this difference is not significant (*P* = 0.56).

### Transition from F0 to F1

The adults F0 were continuously laying eggs, therefore the larvae medium contained larvae of different stages (F1). The first cocoons from F1 were observed in the second week of arrival in the insectary, especially from the larvae collected from Berlese–Tullgren device. The number of newly formed cocoons collected weekly increased until reaching a maximum in week 4 (*n* = 261, Fig. 3). Newly spun cocoons were collected until week 9, for about 915 cocoons in total.

The first emergence of adult F1 was observed on week 3, which is approximately 7 days after the observation of the first cocoons (Fig. 3). Sporadic emergences were recorded daily in weeks 3 and 4, and these adults were collected as they emerged and grouped in a single new container (group 4). Starting from week 5, adults emerged in a larger batch approximately the same day and assembled in a new container (group 5). The maximum emergence of adult fleas was recorded in week 6, and three groups of newly emerged adults of the same age were formed in separate containers (groups 6–8, Additional file 2: Fig. S2). The last emergence was recorded in week 10. Approximately 740 adults F1 were collected from the cocoons, which gave an emergency ratio of approximately 0.8.

### Survival of adults F1 and transition to F2

Flea survival in the F1 cohort was monitored across multiple groups by recording the number of dead individuals at various timepoints (Table 2). The median survival time for F1 cohort was 7 days (95% CI 6–7), although survival times varied significantly between groups, ranging from 6 to 14 days (Table 2). The F1 survival probability is significantly low when compared with F0. The Cox proportional hazards model reveals a significant difference between the two groups (*χ*^2^ = 62.59, *P* = 3e−15). Fleas from generation F1 had a 2.25 times higher risk of dying compared with fleas from generation F0 (HR 2.25, 95% CI 1.82–2.79, *P* < 0.001).

**Table 2:** Number dead and censored *P. irritans* of F1 generation

Group	Collection week	Dead	Censored^a^	Median survival (day)
4	3 and 4	–	–	–
5	5	39	0	14
6	6	267	23	6
9	7	44	12	13
10^b^	8	44	0	9
11^b^	8	28	4	13
12	8	46	0	14
13	9 and 10	20	1	7

^a^ Censored: individuals that survived at the end of the observation time or before merging groups

^b^ Groups 10 and 11 were used to assess the impact of blood source and were not included in the calculation of median survival rate for the F1 population

**Fig. 5:**
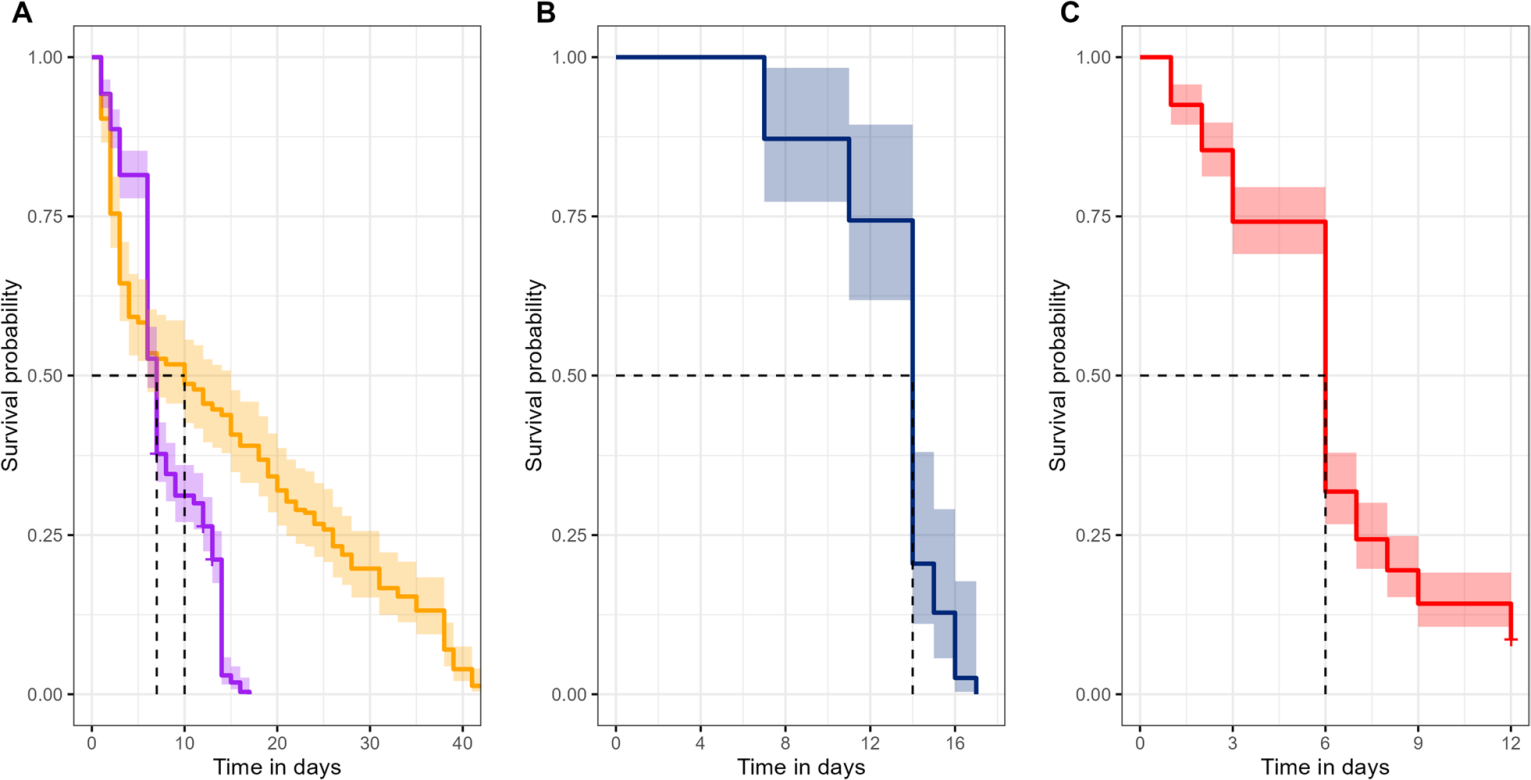
F0, and different groups of F1 survival probabilities. **A** Comparison of survival probability between generations F0 (yellow) and all groups for F1 (purple). The F1 curve is heavily influenced by a simultaneous massive die-off in group 5 (**B**) and group 6 (**C**). Dotted lines show the median survival for each group. Time is in days spent in the insectary

An unusual massive die-off event could have contributed to the overall survivorship trend for F1, as illustrated in Fig. 5A. Notably, group 5 and group 6, which together accounted for more than 72% of the entire F1 population, experienced a substantial die-off on the same calendar day. This occurred on day 14 for group 5 and day 6 for group 6 (Fig. 5B and C), with half of the individuals in each group dying on these days. Cocoons of the F2 generation were observed 10 weeks after the initial arrival of the insects in the insectary, approximately 6 weeks after the emergence of the first F1 progenitors.

### Effect of the blood source on the survival

**Fig. 6:**
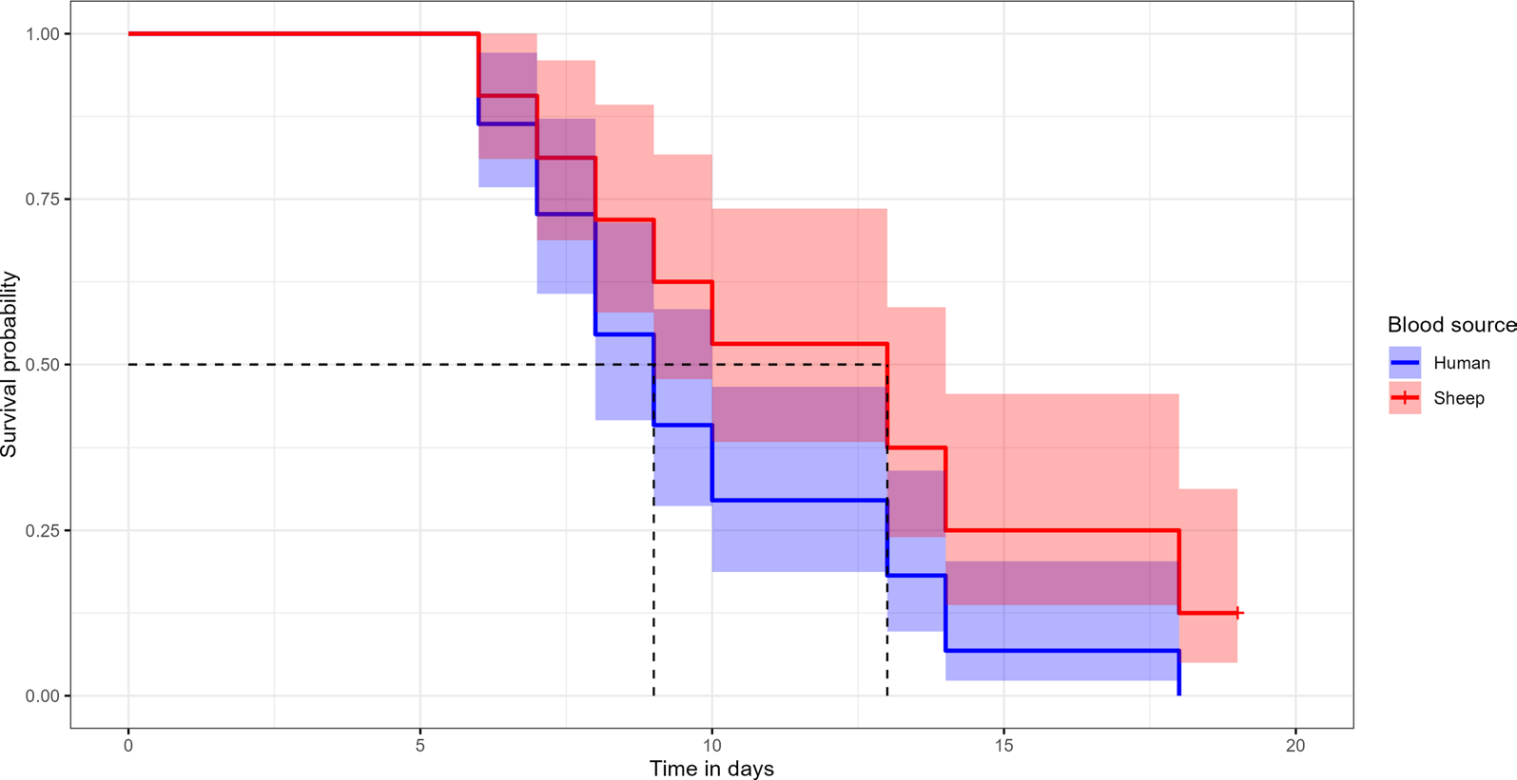
Effect of blood source on survival of F1 generation. Time is in days

Flea survival was significantly influenced by blood source (Fig. 6). The median survival was 9 days for fleas fed with human blood and 13 days for those fed with sheep blood (Table 2). As indicated by the Cox proportional hazards model (*χ*^2^ = 7.13, *P* = 0.008), fleas fed on sheep blood had a 48% lower risk of dying compared with those fed on human blood (HR 0.52, 95% CI 0.32–0.85, *P* = 0.009). However, the concordance index (C = 0.579) suggests that the model has weak-to-moderate predictive power. Still, the survival of adult F1 fed with sheep blood is still poorer when compared with the F0s.

## Discussion

While further studies are needed to establish a sustainable laboratory colony, we demonstrated that collecting both adult and immature stages of *P. irritans* enables the successful rearing of an F1 generation for experimental studies within 3 weeks. This finding highlights the feasibility of working with pathogen-free population for laboratory experimental studies such as infection and transmission experiments or insecticide susceptibility monitoring.

Although adult emerging from BT-collected dust represented a smaller portion of the total F1 population in our study, this method shows promise as a standalone strategy, especially if environmental sampling is intensified and better targeted [38]. It reduces reliance on community participation for adult flea collection, easing ethical constraint. Focusing on immature stages may also help avoid the challenges of maintaining adults via artificial feeding in the field. To maximize BT yield, a preliminary sampling using tools such as candle traps can help to identify households with high flea infestation [25]. Additionally, further adjustment of the BT design may improve larval recovery [38]. In this study, we chose to collect both adult fleas and immature stages to maximize the likelihood of obtaining sufficient F1 generation for laboratory rearing. Adult fleas provided an immediate source for reproduction, while immature stages collected with the BT device offered a valuable backup when adult yields are inconsistent or low. This dual approach increases overall colony success and reduces reliance on a single method.

Our rearing technique was largely inspired by the work of Hudson and Prince, in which adult *P. irritans* were continuously confined inside “pillboxes” and maintained using an artificial feeding system [30]. We kept the blood at 37.5 °C, and successful feeding was confirmed by the presence of abundant blood stains inside the rearing container. Bacot observed that *P. irritans* rapidly consumes a large volume of blood, reaching a point where it must expel waste within a short time, as evidenced by its semi-fluid, bright red excreta [32]. Previous studies reported that *P. irritans* laboratory colonies were fed for 15–30 min, several times daily [30, 32]. In contrast, using the Hemotek system, we achieved good results by allowing feeding for 4–5 h per day every day, significantly reducing handling time. However, as shown in Fig. 5, the sharp decline in the F1 population was likely due to feeding restrictions over a weekend, with high mortality recorded the following Monday. Although the effect of feeding frequency was not directly tested, these findings suggest that frequent access to blood is crucial for the survival and reproduction of *P. irritans*.

The number of adults per group (feeding chamber) was limited to around 80 to avoid overcrowding. Therefore, around weeks 6–7 post-arrival in the insectary, several groups were maintained and fed in parallel with several units of the Hemotek feeder. As the number of fleas in some groups dwindled, the groups were merged to minimize the handling of multiple containers (Additional file 2: Fig. S2). Although this reduced the workload, it complicated the tracking of survival times for individuals from different groups emerging at different times but merged as one group. To calculate the median survival time in each group, we considered the period from the first appearance of adults in the pupae container (group formation) to the last recorded deaths. If the groups were merged before the final deaths occurred, surviving adults were censored at the point of group merging (Table 2).

Fleas from generation F0 had a 56% lower risk of dying compared with fleas from generation F1 (Fig. 5A). Although F0 were transported from the field, they may have survived better in the lab than F1 individuals due to prior exposure to natural selection, which could enhance resilience. Larval diet composition has been shown to significantly impact larval and pupal survival in other flea species [42, 43]. We used a larva diet containing dry sheep blood, yeast, and dog chow, which allowed for the successfully raising of different flea species [30, 39, 44, 45]. F0 adults originated from multiple households, where varying diet and environmental conditions may have influenced their early development. Therefore, testing the impact of different larva diets composition may help to pinpoint the limitation affecting adult F1 survival.

The nature of host blood is known to influence survival and reproduction of *P. irritans*. Although *P. irritans* is considered to be an opportunistic species, feeding on various hosts, Bacot [32] reported that attempts to breed *P. irritans* on rats were unsuccessful, yielding no eggs or offspring. In another experiment involving artificial feeding system with rat blood, he observed that fleas had short lifespans (≤ 14 days), likely due to minimal feeding. Furthermore, strains collected on humans had better pupae production when fed on human blood rather than other sources, and fox strains did better on dog blood than human [30]. Sheep has been reported to be among the host of *P. irritans* in other regions [24, 46]. In our study, fleas were collected using human landing catch, suggesting an anthropophilic feeding behavior. Consequently, human blood was maintained as the principal source for colony maintenance.

Nonetheless, our results unexpectedly showed that the human-derived strain of *P. irritans* had improved survival when fed on sheep blood (Fig. 6). This finding contrasts with previous reports where human-collected strains performed better when fed on human blood [30], and other host-specific strains showed optimal development on their respective hosts [47]. The results should be interpreted with caution due to the low predictive power of our model, likely to stem from limited sample sizes and potential confounding factors. Fleas feeding on their preferred host typically engorge more rapidly, expend less energy on blood digestion due to greater compatibility, and exhibit longer survival under starvation conditions compared with those feeding on nonspecific hosts [1, 47]. This raises important questions about blood-host compatibility and its effect on colony performance.

Given the practical advantage of access to sheep blood in our setting, its potential as a long-term blood source for the colony merits further investigation. As observed with strictly anthropophilic parasites such as body lice, it is possible to adapt strains to feed successfully on laboratory animals such as rabbits [34, 36]. Similarly, sustained efforts to adapt the F1 generation of *P. irritans* to sheep blood are needed. While it remains unclear whether sheep blood leads to reduced feeding performance in *P. irritans*, such as lower engorgement rates, longer feeding, and digestion durations, the use of phagostimulants, including nucleoside triphosphates, could enhance feeding efficiency and acceptance of nonprimary host blood [48]. A gradual host transition protocol should be tested by feeding fleas with mixed blood meals or sequential exposure where fleas will be fed alternatively with human and sheep blood. Indeed, in addition to mortality assessment in larger groups, the effect of blood source on egg or pupae production per group should be monitored as conducted elsewhere [30, 49].

The impact of blood source on arthropod fitness is not unique to *P. irritans* or Siphonaptera. A study showed that both host blood sources significantly affected reproductive success in *Ctenocephalides felis*, with bovine and ovine blood yielding higher egg production under specific male-to-female ratios [49]. Similarly, Vantaux et al. [50] found that consecutive blood meals from different hosts significantly altered mosquito survival, fecundity, and vectorial capacity. In fleas, the influence of blood source on transmission efficiency has also been observed: the ingestion of rat blood by rodent fleas enhanced early phase transmission of *Y. pestis* through regurgitative mechanisms [18, 51, 52]. While these findings concerned other species, and the impact of blood source on *P. irritans* vector competence has been partially addressed [18], further studies are needed. Future research should aim to determine these hypotheses directly in *P. irritans*, investigating whether blood sources not only affect the survival and fecundity of *P. irritans*, but also its ability to acquire and transmit pathogens.

## Conclusions

In this study, we focused on obtaining sufficient *P. irritans* F1 generation for laboratory experiments. While achieving a sustainable laboratory colony remains a challenge, our results highlight key factors influencing flea survival and reproduction. Future studies should focus on optimizing permanent colony maintenance with more accessible blood sources such as sheep blood. Furthermore, the investigation of reproductive parameters such as egg production, hatching rate, and emergence rate, in addition to survival, may help to identify the best conditions to sustain a larger-scale colony.

## Supplementary Information

Additional file 1. Figure S1. Photos of materials developed during this study and used for *P. irritans* collection and rearing; **A** Berlese–Tullgren device; **B** adult/pupae container; **C** adult and larvae containers; **D** adult container inside the larvae container; **E** Hemotek feeding unit showing the parafilm membrane and blood; **F** Hemotek placement during feeding sessions.

Additional file 2. Figure S2. Timeline of adult *P. irritans* group management. Each rectangle in the timeline (blue: F0; green: F1) represents a group of adult *P. irritans* assigned to a single Hemotek feeder unit per day. Group numbers correspond to the chronological order of their creation. Beginning in week 2, the formation of F1 cocoons was monitored and newly formed cocoons were collected weekly from each F0 group and transferred to a designated pupae container. Emerged adults from these containers were collected weekly and assigned to a new group on the basis of their emergence week. For the F1 generation, each group thus consisted of fleas that emerged within the same week. Group sizes were maintained at approximately 80 individuals to avoid overcrowding. Right braces in the timeline indicate instances of groups merging, which served to reduce the number of active feeding chambers when live flea numbers dropped below 10–15 per group. These merging points also represent the censoring times for survival analyses. For example, at week 6, six groups were active, and by week 7, groups 4 and 5 were merged into group 4a to streamline colony management. Notably, groups 10 and 11 were used for the blood source comparison experiment.

## Data Availability

All data generated or analyzed during this study are included in this article and its supplementary materials.

## Abbreviation

BT: Berlese–Tullgren
