# Supplementary figures and images for: Establishing a laboratory colony of the human flea, Pulex irritans: methods for collecting, rearing, and feeding
Source: Parasit Vectors. 2025 Aug 27;18:363. doi: 10.1186/s13071-025-07001-9 (PMC12392626; doi:10.1186/s13071-025-07001-9)

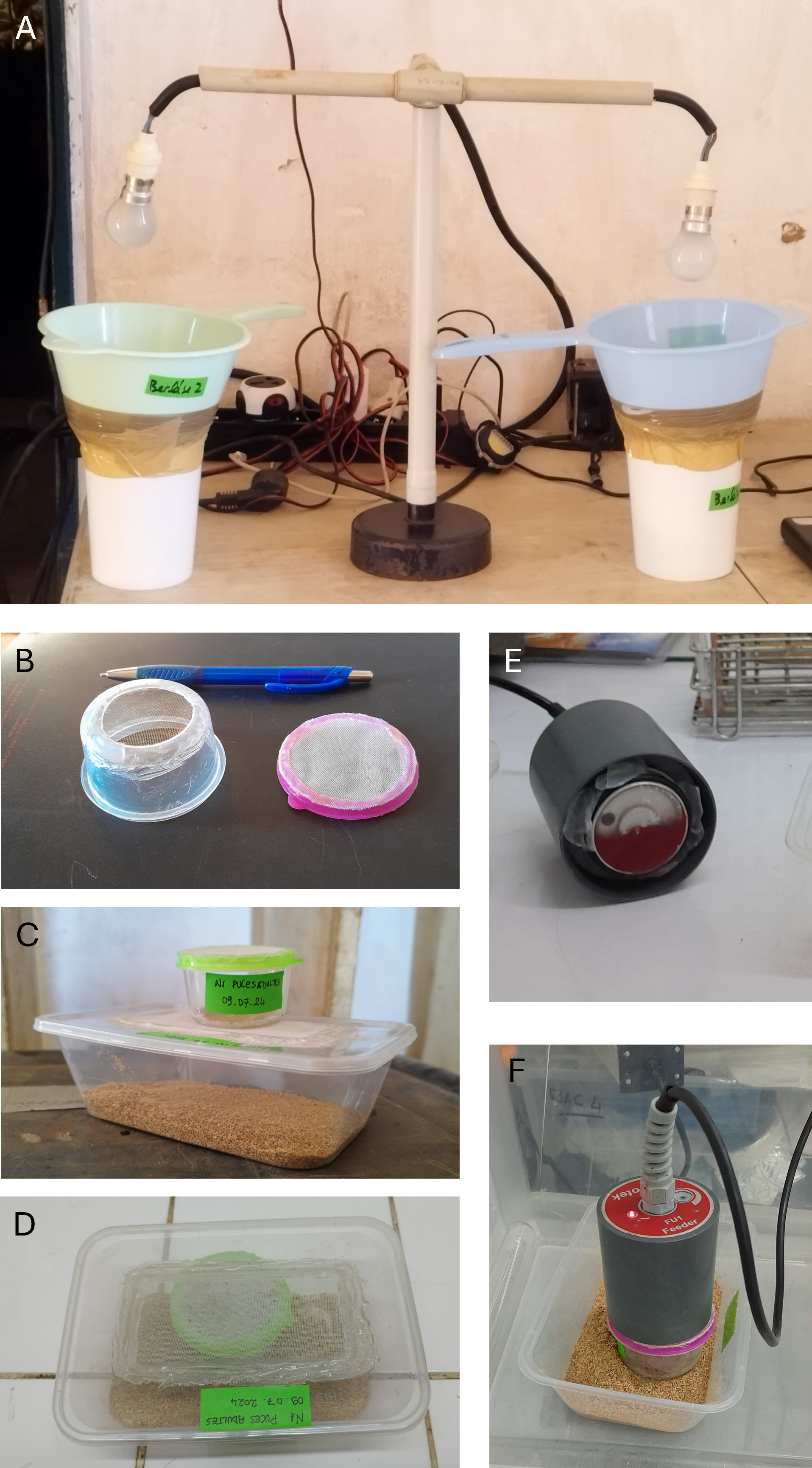

Supplement: Supplementary file 1 — Additional file 1. Figure S1. Photos of materials developed during this study and used for P. irritans collection and rearing; A Berlese–Tullgren device; B adult/pupae container; C adult and larvae containers; D adult container inside the larvae container; E Hemotek feeding unit showing the parafilm membrane and blood; F Hemotek placement during feeding sessions. [file 13071_2025_7001_MOESM1_ESM.tif]

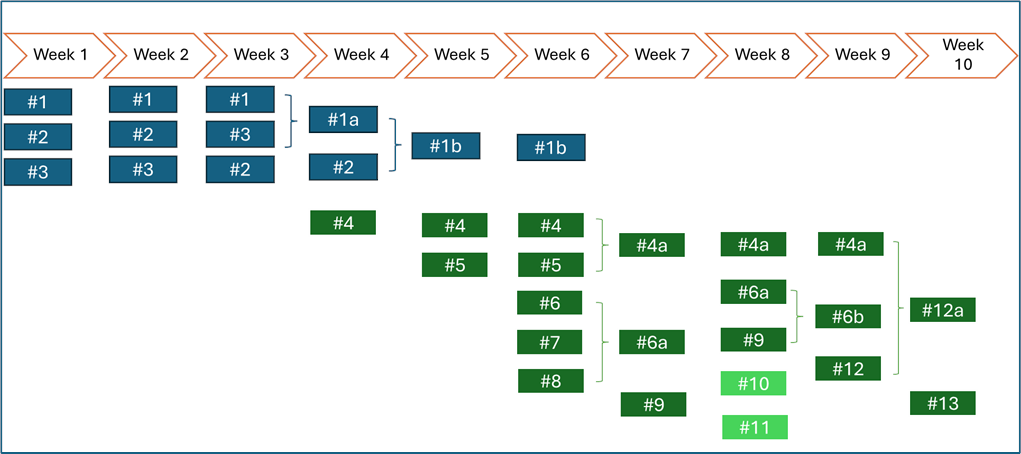

Supplement: Supplementary file 2 — Additional file 2. Figure S2. Timeline of adult P. irritans group management. Each rectangle in the timeline (blue: F0; green: F1) represents a group of adult P. irritans assigned to a single Hemotek feeder unit per day. Group numbers correspond to the chronological order of their creation. Beginning in week 2, the formation of F1 cocoons was monitored and newly formed cocoons were collected weekly from each F0 group and transferred to a designated pupae container. Emerged adults from these containers were collected weekly and assigned to a new group on the basis of their emergence week. For the F1 generation, each group thus consisted of fleas that emerged within the same week. Group sizes were maintained at approximately 80 individuals to avoid overcrowding. Right braces in the timeline indicate instances of groups merging, which served to reduce the number of active feeding chambers when live flea numbers dropped below 10–15 per group. These merging points also represent the censoring times for survival analyses. For example, at week 6, six groups were active, and by week 7, groups 4 and 5 were merged into group 4a to streamline colony management. Notably, groups 10 and 11 were used for the blood source comparison experiment. [file 13071_2025_7001_MOESM2_ESM.tif]
